# Nasal Discharges.—II

**Published:** 1909-06-12

**Authors:** 


### Diseases of Children.

### NASAL DISCHARGES.—II.

Nasal lotions or Collunaria are applied by spray, syringe, irrigation, medicine dropper, brush, probe and cotton, or absorbent wool. Neither plain water nor strong antiseptics or astringents should be used, except silver nitrate as previously mentioned. Lotions should be ordered of a strength treble that at which they are to be used, so that they can be diluted with two parts of hot water. If ordered at the required strength, then the bottle containing the lotion should be placed in water until the temperature reaches 90° F. Under one year of age, or even up to the third year, lotions should be applied by dropping them into the nostrils with the child lying on the back. For older children, spraying, syringing, and douching or irrigation may be adopted. Forceful methods are liable to cause infection of the Eustachian tubes and middle ears.

*Mode of Syringing.*—Use a glass syringe, holding about one-half ounce, carefully disinfected. Cover the nozzle with a piece of soft rubber tubing, for insertion into the nostril to prevent injury. Place the child on its back with the head turned to one side. Stand behind the head and syringe through the upper nostril, allowing the fluid to flow out through the lower nostril or the mouth, which should be open. The child may be held in the sitting posture with the head well forward and a basin under the chin. Older children can clear the nose by blowing, or a small rubber ball syringe may be used. The stream is directed along the floor of one nostril, while the child breathes deeply through the open mouth, each nostril being syringed alternately. Very little force should be used in any method, for fear of infecting the middle ear. Irrigation should not be used for children under four years of age. Douches must be unirritating. Various lotions may be mentioned. (1) Normal saline; (2) Dobell's solution: sod. bicarb. dr. 1, sod. biborat. dr. 1, glycerin. acid. carbolici drs. 2 (or Listerine drs. 4), water ad oz. 10; (3) equal parts of common salt, bicarbonate of soda, and biborate of soda, one teaspoonful to half a pint of water; (4) sodium chloride gr. 2 to 5, potass. bicarb. gr. 2 to 5, boric acid gr. 5 to 10 to the ounce of water; (5) sod. bicarb., sod. bibor. aa. gr. 3, ac. carbol. gr. 1, sugar gr. 5 or glycerin ♏ 20, to the ounce of water; (6) sod. biborat. gr. 10, sod. sulphat. gr. 5 to one ounce of water; (7) one part each of sod. bicarb., sod. biborat., pot. chlorat., and two of white sugar, one teaspoonful to half a pint of water. As an astringent lotion, sulphocarbolate of zinc. grs. 5 to the ounce, is as good as any. It can be used as a spray or with a dropper. Hazeline, 20 drops to the ounce, may be used, or a powder of salicylic acid gr. 3, tannic acid 1 dr., boric acid 1 oz. Passive hyperæmia is also recommended. A flannel bandage, damped with a spirit lotion to ensure shrinking, is applied somewhat tightly round the neck.

2. *Rhinitis due to adenoids* causes a chronic, irritating discharge, generally mucoid, sometimes muco-purulent and sanious. Occasionally it is unilateral. Sometimes it is continuous, more often it varies from time to time. The discharge and the excoriation of the external nares may be the only indications of the presence of adenoids. Eczema, impetigo, swelling of the upper lip, and epistaxis are not infrequent sequelæ. The condition is really a simple chronic rhinitis or post-nasal catarrh, a variety of snuffles, often occurring in acute and recurrent attacks, and its true causation is liable to be overlooked.

3. *Snuffles*, due to congenital syphilis, comes on later in life than the acute purulent rhinitis of infancy and the acute coryza so apt to develop in the newborn. Infection at birth produces it in a day or two. Congenital syphilis causes snuffles in two or three weeks, and a family history or other evidence of the disease may be obtainable. Many cases of simple coryza in infancy are wrongly ascribed to syphilis. The inflammation is usually sub-acute, chronic and persistent, with muco-purulent discharge, nasal obstruction, thickening of the mucous membrane, and the formation of crusts with raw mucous membrane underneath. It may be acute, and followed by sloughing of the mucous membrane and separation of bone. Later in childhood congenital syphilis causes gummatous periostitis and necrosis of cartilage and bone, ulceration, and foul ozæna. Use yellow oxide of mercury ointment (1 in 8).

4. *Acute purulent rhinitis of infancy* is due to infection at birth, by gonorrhœal or leucorrhœal discharge. It comes on very shortly after birth, with redness, swelling, œdema, and a purulent discharge, and prevents sucking. In later life it may be due to gonococcal or pneumococcal infection, and may occur in measles. Cleanse with alkaline lotion and paint with protargol, 1 to 2 per cent., or nitrate of silver, 1 per cent.

5. *Nasal diphtheria*—pseudo-membranous, membranous, or fibrinous rhinitis, is difficult of classification, for some of the cases are apparently diphtheritic, while others cannot definitely be proved due to this cause. Nasal diphtheria may be characterised by mild signs, although there is much membrane, perhaps only visible with a speculum. Occasionally there is no membrane, though pure cultures of the typical bacillus can be obtained. The organism does not seem to produce enough toxins to cause severe symptoms, and the general health is little impaired. It is rare under three years of age, but 90 per cent. are under ten. Often it follows scarlatina. There are pallor, languor, anorexia, and fever up to 100° F., with clear, watery, acrid, irritating nasal discharge, unilateral in one-fifth of the cases. It causes redness and soreness of the alæ and lip, sometimes slight bleeding, and nasal obstruction. Later the discharge becomes purulent, may contain shreds, but is not often offensive. The mucosa is congested, covered with a white film, or a gelatinous, tough, yellowish membrane. It rarely spreads to the larynx or pharynx, but may cause otitis. Finally only blood and muco-pus are exuded. It lasts from one to three months and is not followed by paralysis.

